# Diversify and conquer: How effector diversity is shaped by host–microbe co-evolution

**DOI:** 10.1371/journal.ppat.1013870

**Published:** 2026-01-23

**Authors:** Marion C. Müller, Sabine Brumm, Yiheng Hu, Eric Kemen, Thomas Lahaye, Ralph Hückelhoven

**Affiliations:** 1 Chair of Phytopathology, TUM School of Life Sciences, Technical University of Munich, Freising-Weihenstephan, Germany; 2 Centre of Plant Molecular Biology (ZMBP), Eberhard-Karls-University of Tübingen, Tübingen, Germany; University of Tübingen: Eberhard Karls Universität Tübingen, GERMANY

Effectors are diverse molecules produced and secreted by plant pathogens to facilitate infection often by exerting an “effect” on their host. The production of effector molecules is a ubiquitous feature of plant pathogens. Because many effector proteins act on host targets and are subject of host immune detection, the corresponding effector genes co-evolve with the corresponding host genes involved in immunity or susceptibility. This co-evolution results in diversity at effector loci, both within and between lineages/species. Here, we examine the diverse patterns and consequences of variation observed across the sequence and regulatory landscapes of effector genes and summarize the genomic mechanisms that create them.

## 1. What are effectors?

Effectors act on their corresponding host through various molecular functions, including the interference with the host immune system, facilitation of nutrient uptake, or promotion of pathogen proliferation [1]. While effectors are most commonly proteins, others take the form of small RNAs, such as those involved in trans-kingdom RNAi, or low molecular components like secondary metabolites. All kingdoms of plant pathogens, including bacteria, fungi, oomycetes, viruses, and parasites such as nematodes produce effectors, albeit in variable numbers. For instance, obligate biotrophic cereal rust fungi can encode over 1,000 effectors, whereas plant–pathogenic bacterial genera *Pseudomonas* and *Xanthomonas* typically encode only 20–40 effectors [2,3]. In this review, we primarily focus on protein effectors, as they represent the most well-characterized class of effectors to date. Pathogens employ different strategies to ensure that effectors reach their place of action either in the host’s intracellular or apoplastic space. In fungi and oomycetes, this is achieved in many cases through the secretory pathway [4]. Therefore, the presence of a signal peptide is often used as a criterion to identify fungal and oomycete effectors, although some evident effectors lack secretory leader signals [5,6]. In bacteria, effectors are usually exported via specialized transport systems—known as Type II or III secretion systems—which facilitate their export and/or direct translocation into host cells [7].

## 2. Why are effectors diverse?

There are two reasons why effectors diversify during evolution: either to evade recognition by the plant immune system or to adapt to their host targets. Plants encode a wide array of immune receptors that recognize different pathogen molecules, including effectors, which are then referred to as avirulence proteins. Consequently, plants impose strong selection pressure on pathogens to avoid recognition. When new mutations in effectors occur in a population, either as de novo events or from standing genetic variation, natural selection can act on these variants, allowing them to increase in frequency if they confer a fitness advantage. A tangible example of pathogen adaptation is the rapid resistance breakdown frequently observed in agricultural systems, due to increase in effector variants that evade detection by the plant [8]. The increase in frequency of a beneficial mutation, known as a selective sweep, can be detected in population-level genomic data because it causes a local reduction in genetic diversity around the advantageous mutation. This pattern was, for instance, observed for the *AvrPm17* gene in certain wheat powdery mildew populations in Europe [9]. Another measure for detecting selection at the population level is the ratio of nonsynonymous to synonymous substitutions (dN/dS), as recurrent selective sweeps lead to an excess of nonsynonymous changes, as was observed in certain effectors of *Magnaporthe oryzae* [10].

Several models describe the co-evolution of effectors and their hosts' receptors. The gene-for-gene model emphasizes the reciprocal matching between host resistance genes and pathogen avirulence genes, where a change in one partner (e.g., the host receptor) drives a corresponding change in the other (e.g., the effector) [11]. Two widely used models for co-evolution between effectors and resistance loci are the arms race and trench warfare dynamics (reviewed in [12]). Under arms_race dynamics, polymorphisms at effector loci are typically transient because recurrent selective sweeps fix variants that confer a fitness advantage to the pathogen. In contrast, trench_warfare dynamics maintain polymorphisms at effector loci, leading to the coexistence of virulence and avirulence alleles in the population and signatures of balancing selection. Whether co-evolution follows an arms race or trench warfare dynamics depends on multiple factors, including the type of selection and the fitness costs associated with virulence and resistance, as well as the heterogeneity of selection (reviewed in [12]). Generally, arms race dynamics are considered more likely to occur in agricultural systems, whereas trench warfare dynamics are expected to occur in natural systems.

While population genetics have focused on generating evolutionary frameworks that explain effector diversification, molecular biology has focused on unraveling the molecular mechanisms that lead to diversification of effectors. For instance, the wheat resistance gene *Sr35*, which encodes an intracellular receptor of the NB-LRR (nucleotide-binding, leucine-rich repeat) class, directly binds the avirulence effector AvrSr35 from the stem rust pathogen *Puccinia graminis f. sp. tritici* (*Pgt*) [13,14]. However, certain isolates evade recognition by Sr35 due to the insertion of a transposable element into the AvrSr35 coding sequence, which introduces a premature stop codon and renders the protein non-functional in triggering immunity [15].

A well-described example for effectors that adapt to their host target are the Transcription Activator-Like Effectors (TALEs) from *Xanthomonas* spp. (leaf-infecting bacteria), which share a modular DNA-binding domain composed of 15–20 tandem repeats, each 33–35 amino acids in length [16]. Each repeat binds a single base in a contiguous target sequence, known as the effector binding element, which is located upstream of host target genes and mediates transcriptional activation. Base preference of each repeat is determined by the repeat-variable diresidue (RVD) at positions 12 and 13. In TALEs, the non-RVD residues are highly conserved at both amino acid and nucleotide levels, with each position—such as residue 2 or 10—occupied by the same amino acid and encoded by the same codon. This position-specific conservation occurs both within a single TALE and across different TALEs. Such homogeneity enables intra- and intermolecular repeat rearrangements, supporting the idea that some TALEs may serve as repeat reservoirs to promote sequence diversification through modular shuffling [17].

Pathogen effectors not only adapt to host targets but are also, in turn, targeted by host countermeasures, triggering a new round of adaptation. The interaction between fungal polygalacturonases (PGs) and plant polygalacturonase-inhibiting proteins (PGIPs) is a prominent example for this. A recent study showed that a PG from *Fusarium phyllophilum* is not only bound and inhibited by PG-binding PGIP2 from *Phaseolus vulgaris*, but that this protein-protein interaction forces the complex into a changed enzyme activity that creates long- rather than short-chain oligogalacturonides. Importantly, while short-chain oligogalacturonides are immune-suppressive, longer-chain oligogalacturonides function as elicitors that activate plant immunity [18]. Similar to plant immune receptors, PGIPs contain leucine-rich repeats for PG interaction, and target binding amino acids in PGs have been shown to be under diversifying selection. A picture thus emerges, in which PGIPs evolved as potent host tools to take advantage of a pathogens’ pathogenicity factor in generating immune-active instead of immune-suppressive elicitors. However, the pathogen might adapt to this by changing its PG surface composition, making the PG less accessible to the plant PGIP. PGIPs again appear to adapt to this either by amino-acid changes within PGIPs or by gene family copy number variation for tailoring new isoforms [19].

## 3. What kind of diversity exists among effector genes and how is it created?

**Fig 1:**
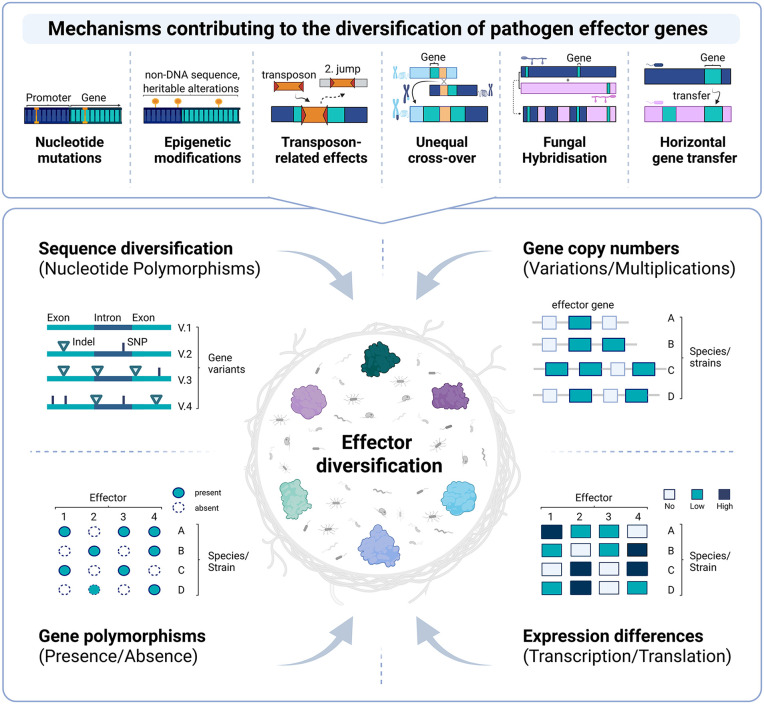
Schematic representation of the most important mechanisms that create diversity in effector genes (top) and different patterns of diversity observed at effector loci (bottom). Created in BioRender. Brumm, S. (2026) https://BioRender.com/m6wj203.

There are four main patterns of diversity commonly in effector genes that are readily reported in the scientific literature (Fig 1). First, pathogen effectors often exhibit **sequence diversity**, which includes single-nucleotide polymorphisms as well as insertions and deletions. Sequence variation that leads to amino-acid exchange can diversify protein interaction surfaces, enabling differential interactions with host targets and immune receptors [20]. Second, effectors often exhibit **presence/absence** polymorphisms, where certain genes are missing in specific isolates or lineages [21]. Third, effectors can also experience higher-order **copy number variation** through duplication of effectors that then can undergo further diversification. Finally, diversity can also occur at the **gene regulatory** level leading to differences in effector doses [22].

The molecular mechanisms that create these different levels of effector diversity are well understood. For instance, **mutation**, such as point mutations, insertions, and deletions, which alter the nucleotide composition of both coding and non-coding regions of effector genes (Fig 1) [23]. **Recombination** during meiosis (crossover) allows pathogens to reshuffle their effector repertoires. In contrast, unequal crossover between misaligned homologous DNA sequences results in an unequal exchange of genetic material which leads to duplications and deletions. Such events are major drivers of copy number and presence/absence variation [24] (Fig 1). Unlike mutation or recombination, epigenetic changes do not alter effector DNA sequences but regulate expression through mechanisms such as chromatin remodeling and histone or DNA modifications. For instance, the *Phytophthora sojae* effector gene *AvrPm1b* was shown to be transcriptionally silenced by histone methylation, a mark typically associated with gene repression in eukaryotes [25]. **Transposable elements (TE)** are mobile DNA elements, that often as part of their attempt to replicate and insert themselves into their host’s genome, create a special case of insertion mutation. Such insertions into the coding region are often disruptive and insertion into regulatory elements can alter effector expression (Fig 1). Alternatively, host defence mechanisms that target and silence TEs via methylation or mutation may also affect nearby effector genes [26]. **Horizontal gene transfer** (HGT), the exchange of genetic material between sexually incompatible organisms, also contributes to effector diversity (Fig 1). For instance, the fungal toxin effector ToxA, which induces cell death in wheat lines carrying the corresponding susceptibility gene *Tsn1* [27], was transferred by HGT mediated by a giant transposable element between three distantly related fungal species. Finally, **hybridization**, the crossing of pathogens from different species, can also contribute to effector diversity by reshuffling entire effector repertoires [28] (Fig 1).

## 4. What remains to be discovered about effector diversity in plant pathogens?

Several aspects of effector diversification remain poorly understood. For example, the same host plants can be infected by multiple pathogens with distinct lifestyles, each employing different modes of effector diversification. On wheat, the obligate biotrophic fungus *Blumeria graminis* possesses large, expanded clusters of highly similar effector gene duplicates [22]. In contrast, *Zymoseptoria tritici*, a hemibiotrophic fungus features a compartmentalized genome with accessory chromosomes and high levels of standing sequence variation in its effector genes [29]. Thus, different effector diversification strategies occur in the co-evolution with the same host. A key unresolved question is what determines the frequency and type of diversification mechanism across different species? For instance, are pathogens with weakened defense systems against repetitive elements more permissive to gene duplication and display higher rates of effector disruption by TE insertion? Additionally, different pathogenic lifestyles likely involve co-evolution with distinct sets of host targets and immune receptors, which may favor different mechanisms of effector diversification. Finally, the lifestyles of pathogens, for example, whether they reproduce sexually or asexually, influence how efficiently selection acts on beneficial effectors and deleterious mutations. Thus, understanding effector diversification requires comparative analyses across diverse pathogen species and lifestyles, coupled with accurate descriptions of diversity and population genomics analysis.

Another aspect that remains poorly understood is the nature of the selection pressures driving effector diversity. In the simplest scenario, where a single immune receptor co-evolves with a single avirulence effector, immune evasive effector variants are expected to increase in frequency if the selection pressure imposed by the receptor is strong enough. This outcome depends on both the frequency of the immune receptor within the host population and the fitness cost associated with effector modification. However, growing evidence suggests that receptor/effector dynamics often involve complex molecular networks rather than simple one-to-one relationships. For example, the wheat Pm4/Rmg8 immune receptor recognizes two distinct avirulence effectors from powdery mildew (*Bgt*) and wheat blast pathogens [30–32]. The *Pm4/Rmg8* immune receptor is suppressed by a *Bgt* effector, which reversely is recognized by another immune receptor [33]. This creates a multilayered interaction network, in which both the effector and the host proteins may co-evolve with multiple molecular partners.

Beyond effector perception by host immune receptors, the role of effector targets in shaping effector evolution remains largely unexplored. Recent pangenomic analyses across various plant species have revealed a high proportion of non-conserved genes within single species [34]. However, the specific diversity occurring at host effector targets remains largely unexplored. Such diversity may impose selection on pathogen effectors, particularly those targeting variable host proteins. Therefore, incorporating co-evolutionary models for effector–effector target interactions, in addition to immune receptors, will likely generate a more comprehensive understanding of effector diversification.

An additional striking layer of complexity is introduced by an increasing number of reports showing that microbial effectors, rather than acting exclusively on host proteins, also function in microbial competition [35]. This observation extends the classical definition of effectors in the microbial pathogenic lifestyle beyond their role in manipulating the host. For instance, fungal pathogens such as *Verticillium dahliae* secrete antimicrobial effectors that suppress competing microbes in the host environment, thereby promoting colonization and virulence. These effectors not only support infection but also actively shape the local microbiota. Recent studies have demonstrated that the selective pressures driving effector diversification extend beyond host immunity to include intermicrobial antagonism [35]. Incorporating this ecological perspective into our models of effector evolution may help explain the coexistence of conserved effectors with rapidly diversified ones.

Finally, the question remains: how do entirely new effectors, which do not originate from gene duplication or HGT, arise in the genomes of plant pathogens? It is often speculated that non-coding regions of the genome serve as a starting material for such innovation through the acquisition of transcriptional and coding potential [36]. Alternatively, TEs, which are abundantly present in genomes of plant pathogens [37], may serve as a template for the de novo evolution of effector proteins. Indeed, several studies report on the evolution of either RNA or protein effectors that originate from TEs and have an influence on the virulence of the pathogens expressing them [38,39]. In addition, novel effectors could also originate from environmental DNA. Despite anecdotal reports of de novo effector evolution, a comprehensive picture of the rates and sources underlying such innovations has yet to be established.

In summary, the mechanisms by which effectors vary in number, expression, and structure remain incompletely understood. Pathogen pangenomes, which allow capturing the existing diversity to its full extent, will likely be instrumental in advancing our understanding of effector diversity. Understanding why certain effectors diversify and others not, may require a better understanding of the selective forces acting on them. To achieve this, advanced insights into the mechanisms of effector diversification derived from genomic data should be combined with population genomics tools to quantify the selective forces acting on effector evolution. A more holistic view considering interactions with multiple host proteins, potential alternate hosts, and microbial competitors in the pathogen’s niche will be key to unraveling effector evolution in its full complexity.
